# Ets1 Controls the Development of B Cell Autoimmune Responses in a Cell-Intrinsic Manner

**DOI:** 10.4049/immunohorizons.1900033

**Published:** 2019-07-17

**Authors:** Alex Sunshine, David Goich, Alifa Stith, Katherine Sortino, Justin Dalton, Sarah Metcalfe, Eric C. Svensson, Lee Ann Garrett-Sinha

**Affiliations:** * Department of Biochemistry, State University of New York at Buffalo, Buffalo, NY 14203;; † Division of Cardiology, Department of Medicine, University of Chicago, Chicago, IL 60637

## Abstract

Ets1 is emerging as a key transcription factor that is required to prevent autoimmunity in mice and humans. Ets1 is expressed in both B and T cells, and mice lacking Ets1 are characterized by excess B and T cell activation, leading to enhanced formation of Ab-secreting cells and high titers of autoantibodies. In humans, genome-wide association studies have detected associations of single nucleotide polymorphisms in the human *ETS1* gene with autoimmune diseases, including lupus. An increased fraction of CD4^+^ T cells from Ets1^−/−^ mice have an activated effector-memory phenotype, and there are aberrations in differentiation that contribute to the autoimmune phenotype. In vitro studies of B cells suggest that Ets1 may have B cell–intrinsic effects as well. To confirm B cell–intrinsic roles for Ets1, we crossed CD19-Cre mice to mice with a floxed allele of Ets1. Mice with a B cell–specific deletion of Ets1 show increases in B cell activation, numbers of Ab-secreting cells, and levels of autoantibodies, despite the fact that T cells are normal. However, when compared with conventional Ets1 knockout mice, mice with B cell–specific loss of Ets1 have a significantly milder phenotype. These results demonstrate that Ets1 is required in B cells to prevent autoimmune responses but that loss of Ets1 activity in other cell types is required for maximal autoimmune phenotypes.

## INTRODUCTION

Autoimmune diseases such as systemic lupus erythematosus result from immune system recognition of and activation by self-antigens. In aggregate, these diseases are thought to affect 5–10% of the population (1, 2). The causes of autoimmune disease are complex and depend on a variety of genetic and environmental factors. One gene implicated in the development and progression of autoimmune diseases is Ets1 (3), which encodes a transcription factor highly expressed in B and T lymphocytes. Ets1 knockout mice develop a lupus-like autoimmune disease, accompanied by aberrant B and T cell differentiation (4–10). The human *ETS1* gene has also been identified as a susceptibility locus for development of lupus and multiple other autoimmune diseases (11–18).

B cells from Ets1^−/−^ mice show a variety of defects including loss of the marginal zone B cell population, increased levels of activation markers in follicular B cells, increased isotype switching to IgG1 and IgE, reduced switching to IgG2a, and increased numbers of Ab-secreting cells (ASCs) (4–6, 19). The increase in ASCs in Ets1^−/−^ mice is accompanied by increases in serum IgM and IgG1 levels and the secretion of autoantibodies that deposit as immune complexes in the kidney glomeruli (4, 5, 8, 19). Transfer of purified Ets1^−/−^ B cells into wild-type hosts results in downregulation of several activation markers including CD23, CD80, and CD86 (4), indicating that part of the B cell phenotype in Ets1^−/−^ mice is B cell extrinsic. T cells from Ets1^−/−^ mice have severe aberrations, including increased differentiation to an effector/memory phenotype and altered differentiation of Th subsets (8–10, 20–22). Freshly isolated CD4^+^ T cells from Ets1^−/−^ mice express high levels of mRNA for IL-4, IL-5, IL-10, and IL-13 but reduced levels of mRNA for IFN-γ (8). Ets1^−/−^ CD4 T cells cultured in vitro under appropriate skewing conditions show similarly reduced IFN-γ production but reduced rather than enhanced production of Th2 cytokines (IL-4, IL-5, and IL-13) (10). Ets1^−/−^ CD4 T cells give rise to increased percentages of IL-17–secreting cells (21). In addition, there are reduced numbers of Foxp3^+^ regulatory T cells (Tregs) in Ets1^−/−^ mice, and the Tregs that do develop express low levels of Foxp3 and are poor suppressors of effector T cell responses (8). Transfer of wild-type Tregs into chimeras containing Ets1^−/−^ T cells results in the restoration of Ets1^−/−^ marginal zone B cells and reduced production of IgG1 and IgE Abs (8). Therefore, impaired Treg function was suggested to be the cause of several B cell defects resulting from the absence of Ets1. However, a more recent study in which Ets1 was specifically deleted in the T cell population shows that the major T cell aberration underlying the autoimmune phenotype of Ets1^−/−^ mice is excess T cell differentiation to T follicular helper cells that secrete IL-4 (T follicular helper type 2 [Tfh2] cells) (20).

Together, the results described above indicate that abnormalities in the CD4^+^ T cell population in Ets1 knockout mice may drive the aberrations in the B cell compartment. However, this does not preclude an important role of Ets1 in B cells and certain data substantiate a function of Ets1 within B cells themselves. For instance, in vitro culture of purified Ets1^−/−^ B cells in the presence of TLR ligands results in hyperproduction of ASCs (4, 23). In addition, retrovirally driven expression of Ets1 in differentiating wild-type B cells prevents their differentiation to ASCs (23–25). B cell–intrinsic functions of Ets1 in regulating ASC generation include the ability of Ets1 to bind to and inhibit the function of the ASC transcription factor Blimp1 (24, 25) and direct regulation of genes important for B cell differentiation (26). Purified Ets1-deficient B cells cultured with LPS and IFN-γ show a defect in switching to IgG2a (5). Furthermore, mixed bone marrow chimeras generated with congenically labeled wild-type and Ets1^−/−^donor cells showed that Ets1^−/−^ B cells give rise to more ASCs than wild-type B cells when they are found together in the same microenvironment (23). In the current study, analysis of mice with a B cell–specific deletion of Ets1 shows that there is a cell-intrinsic role for Ets1 in regulating B cell activation, development of ASCs, and production of autoantibodies. These B cell–intrinsic functions of Ets1 likely cooperate with T cell–intrinsic functions of Ets1 to modulate immune responses.

## MATERIALS AND METHODS

### Mice

Ets1 floxed mice have been reported previously (27) and have been bred to the C57BL/6 genetic background for >12 generations. CD19-Cre mice were obtained from Jackson Laboratory and are on a C57BL/6 background. Conventional Ets1 knockout mice and littermate wild-type controls were bred in our colony and are maintained on a mixed genetic background (C57BL/6 × 129Sv), because of perinatal lethality on a pure C57BL/6 background. Animal experiments were performed under the approval and guidance of the Institutional Animal Care and Use Committee of Roswell Park Cancer Institute.

### Western blotting

B cells were purified from spleens of mice using magnetic beads to select CD43-negative cells, and lysates were prepared. Western blotting was performed using monoclonal anti-Ets1 Ab (Clone D808A; Cell Signaling Technology). Blots were reprobed with monoclonal anti-GAPDH Ab (Clone 6C5; MilliporeSigma) as a loading control.

### Flow cytometry

Single-cell suspensions of spleen and lymph nodes were prepared using standard techniques and then stained with Ghost Dye Violet 510 to label dead cells. Cells were subsequently stained with various combinations of Abs against the following Ags to assess B cell differentiation and activation status: B220, CD21, CD23, CD73, CD80, CD86, CD138, Fas, IgM, IgD, IgG1, and PD-L2 as well as peanut agglutinin (PNA). T cell differentiation was assessed by staining with Abs against CD4, CD8, CD62L, CD44, CD86, and intracellular Foxp3. Flow cytometry data were collected on an LSR II flow cytometer. Flow cytometry data were analyzed using FlowJo software to gate on live (Ghost Dye Violet 510 negative) cells and subsequently gated on singlets based on side scatter area versus width and on the lymphocyte gate based on forward scatter area versus side scatter area. B and T lymphocytes were further gated based on positivity for B220 (B cells) or CD4 (T cells), and expression of relevant markers in these populations was then examined.

### ELISA

Solutions of 10 μg/ml anti-mouse IgM, anti-mouse IgG, or various potential autoantigens were used to coat MaxiSorp 96-well ELISA plates overnight. Autoantigens included mixed calf thymus histones (Sigma-Aldrich), calf thymus dsDNA (ThermoFisher Scientific), mouse purified myelin basic protein (MBP) (Sigma-Aldrich), and Smith Ag (SMA-3000 containing SmB, SmB’, and SmD Ags; ImmunoVision). Dilutions of serum from mice of various genotypes were added to plates, and ELISA was performed using standard techniques and TMB substrate. Absorbances were read at 450 nm. For total serum IgM and IgG, absorbance values were compared with a standard curve of purified mouse IgM or IgG to calculate exact concentrations. For autoantibodies, data are reported as the absorbance values.

### ELISPOT

ELISPOT plates (MultiScreen 96-well plates with Immobilon-P membranes; MilliporeSigma) were coated overnight with anti-IgM (BioLegend) or anti-IgG (SouthernBiotech) Abs. Single-cell suspensions of spleen and lymph node were plated at various dilutions, and ELISPOT assay was performed as previously described (24).

### Staining of kidney sections for immune complex deposition

Kidneys were harvested from mice, frozen in Tissue-Tek OCT medium, and sectioned with a cryostat. Sections were fixed in paraformaldehyde and stained with FITC-conjugated anti-mouse IgM or anti-mouse IgG. Images were captured with a Nikon 80i fluorescence microscope and analyzed using Fiji software.

### Statistics

All data are shown as mean ± SEM. Means between Ets1^+/+^ and Ets1^−/−^ and between CD19-Cre Ets1^+/+^ and CD19-Cre Ets1^fl/fl^ mice were compared using unpaired Student *t* test or Mann–Whitney *U* test depending on the distribution of data. A *p* value ≤0.05 was considered significant.

## RESULTS

### Generation of mice lacking Ets1 specifically in B cells

**FIGURE 1.**
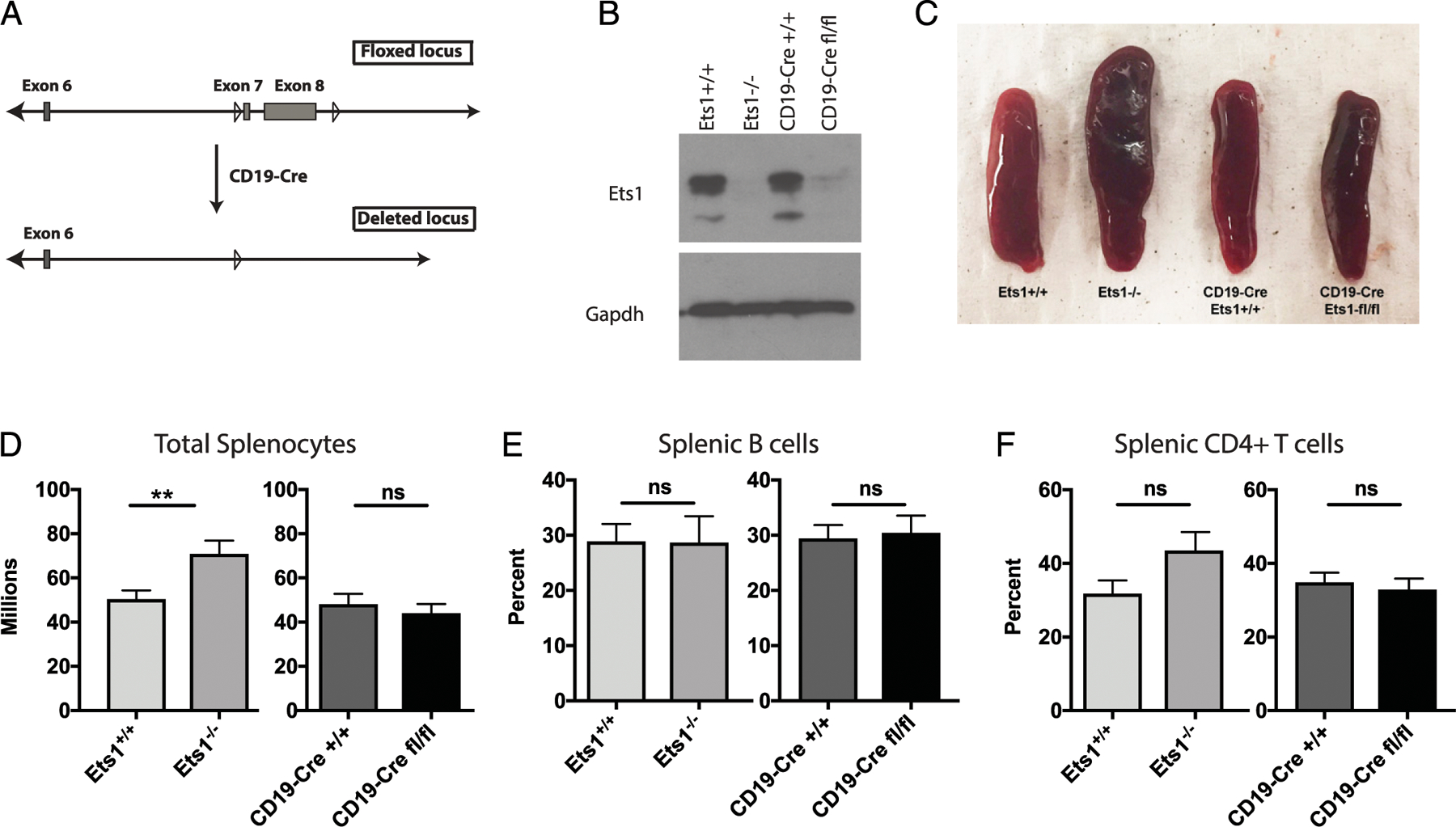
Generation and initial characterization of mice lacking Ets1 in B cells. (**A**) Generation of B cell–specific Ets1 knockout mice. Diagram of the floxed allele (top) in which loxP sites flank the final two exons of the Ets1 gene (exons 7 and 8) and the deleted allele (bottom). (**B**) Western blot for Ets1 protein (top) using lysates from purified splenic B cells. GAPDH was used as a loading control (bottom). (**C**) Spleens from mice of the indicated genotypes showing that Ets1^−/−^, but not CD19-Cre Ets1^fl/fl^, mice have enlarged spleens. (**D**–**F**) Quantification of the number of total splenocytes (*n* = 20 Ets1^+/+^ and Ets1^−/−^ and 12 CD19-Cre Ets1^+/+^ and CD19-Cre Ets1^fl/fl^ mice), splenic B cells (*n* = 8 Ets1^+/+^, 6 Ets1^−/−^, 11 CD19-Cre Ets1^+/+^, and 12 CD19-Cre Ets1^fl/fl^ mice), and splenic CD4^+^ T cells (*n* = 7 Ets1^+/+^, 5 Ets1^−/−^, 10 CD19-Cre Ets1^+/+^, and 8 CD19-Cre Ets1^fl/fl^ mice) in mice of the indicated genotypes. **p < 0.01. ns, not significant.

To test B cell–intrinsic versus B cell–extrinsic roles for Ets1, mice specifically lacking Ets1 in B cells were generated by crossing the well-characterized B cell–specific CD19-Cre line with mice carrying a floxed allele of Ets1 to generate CD19-Cre Ets1^fl/fl^ mice (Fig. 1A). The floxed allele of Ets1 carries loxP sites flanking the last two exons of the gene (exons 7 and 8), which encode the DNA-binding domain of Ets1. Western blotting showed that Ets1 was efficiently deleted in B cells from CD19-Cre Ets1^fl/fl^ mice, yielding levels similar to that found in conventional Ets1 knockout mice (Ets1^−/−^) (Fig. 1B). The spleens of CD19-Cre Ets1^fl/fl^ mice were normal in size, whereas spleens of conventional Ets1 knockout mice were enlarged (Fig. 1C). In keeping with the normal spleen size, the total numbers of splenocytes, splenic B cells, and splenic T cells of CD19-Cre Ets1^fl/fl^ mice were similar to those found in wild-type (Ets1^+/+^) and CD19-Cre only (CD19-Cre Ets1^+/+^) control mice (Fig. 1D–F).

### Loss of Ets1 specifically in B cells results in aberrations in B cell differentiation

**FIGURE 2.**
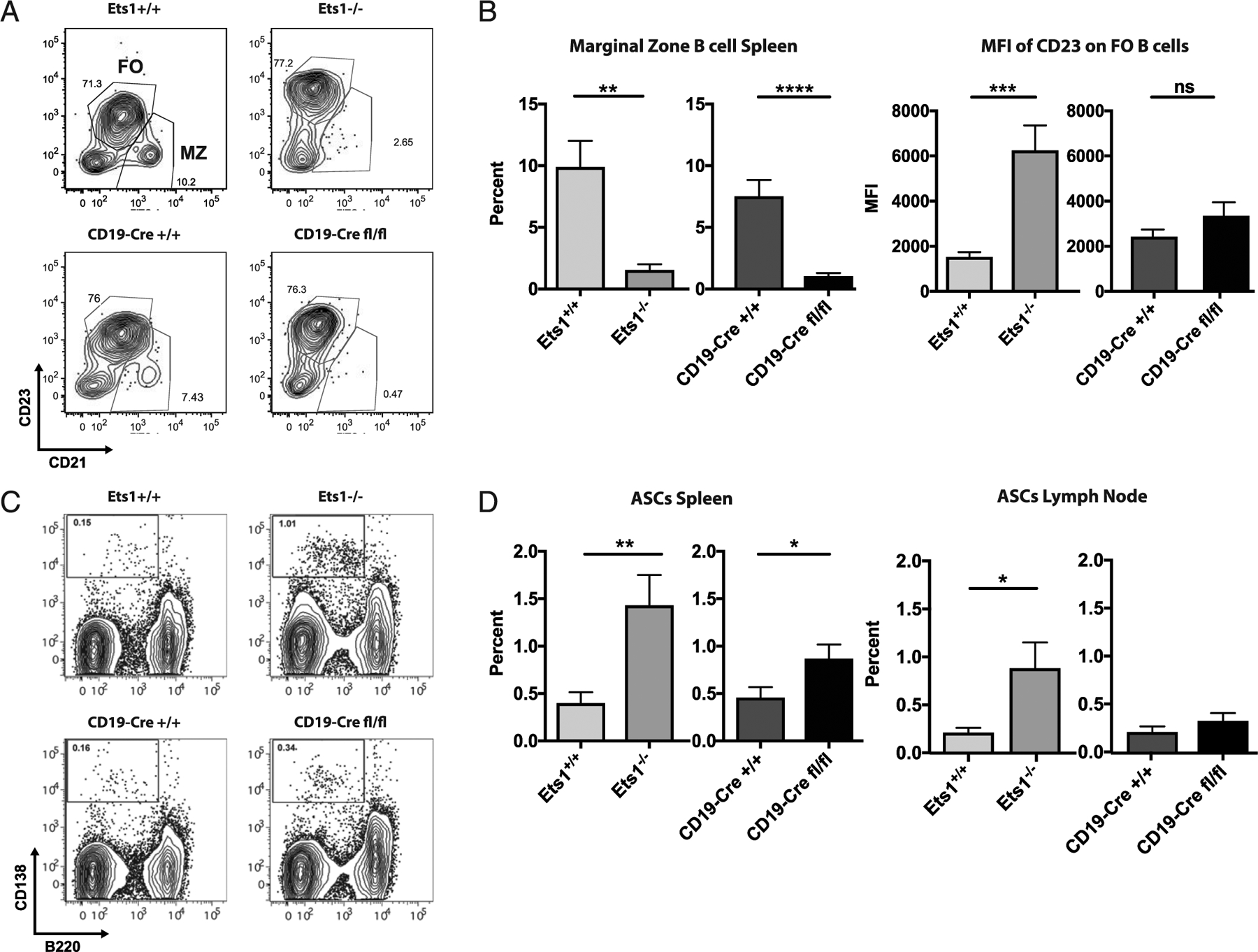
Mice with a B cell–specific loss of Ets1 have reduced marginal zone B cells and increased ASCs. (**A**) Flow cytometry analysis of CD21 versus CD23 in gated live, B220^+^ B cells showing that Ets1^−/−^ and CD19-Cre Ets1^fl/fl^ mice lack CD21^hi^CD23^lo^ marginal zone B cells. Gate positions were adjusted to enclose the major cell populations. (**B**) Quantification of the percent of marginal zone B cells and the mean fluorescence intensity (MFI) of CD23 on follicular B cells (*n* = 8 Ets1^+/+^, 6 Ets1^−/−^, 11 CD19-Cre Ets1^+/+^, and 8 CD19-Cre Ets1^fl/fl^ mice). (**C**) Flow cytometry analysis of B220 versus CD138 in gated live cells showing that Ets1^−/−^ and CD19-Cre Ets1^fl/fl^ mice have increased percentages of B220^lo^CD138^+^ Ab-secreting plasma cells. (**D**) Quantification of the percent of ASCs in the spleen and lymph nodes of the various strains of mice (*n* = 7 Ets1^+/+^, 6 Ets1^−/−^, 9 CD19-Cre Ets1^+/+^, and 11 CD19-Cre Ets1^fl/fl^ mice). *p < 0.05, **p < 0.01, ***p < 0.001, ****p < 0.0001. ns, not significant.

Conventional Ets1^−/−^ mice have a loss of marginal zone type B cells and a higher than normal expression of CD23 on follicular B cells (Fig. 2A–C). CD19-Cre Ets1^fl/fl^ mice also lack marginal zone B cells (Fig. 2A, 2B), whereas the levels of CD23 on follicular B cells from CD19-Cre Ets1^fl/fl^ mice were not elevated (Fig. 2A, 2B). Like Ets1^−/−^ mice, CD19-Cre Ets1^fl/fl^ mice have an increase in B220^low^CD138^+^ ASCs in the spleen, although the overall percentages and numbers of ASC were not as high in CD19-Cre Ets1^fl/fl^ mice as in Ets1^−/−^ mice. Furthermore, CD19-Cre Ets1^fl/fl^ mice did not have an elevation of ASC in their lymph nodes (Fig. 2C, 2D), despite the fact that Ets1^−/−^ mice do. Because not all ASCs express CD138 (28), ELISPOT was performed to enumerate both IgM- and IgG-secreting cells in the spleens and lymph nodes of CD19-Cre Ets1^fl/fl^ and control mice. The number of IgM-secreting ASCs was elevated in the spleens and lymph nodes of CD19-Cre Ets1^fl/fl^ mice, whereas IgG-secreting cells were only significantly elevated in the lymph nodes of CD19-Cre Ets1^fl/fl^ mice (Supplemental Fig. 1).

**FIGURE 3.**
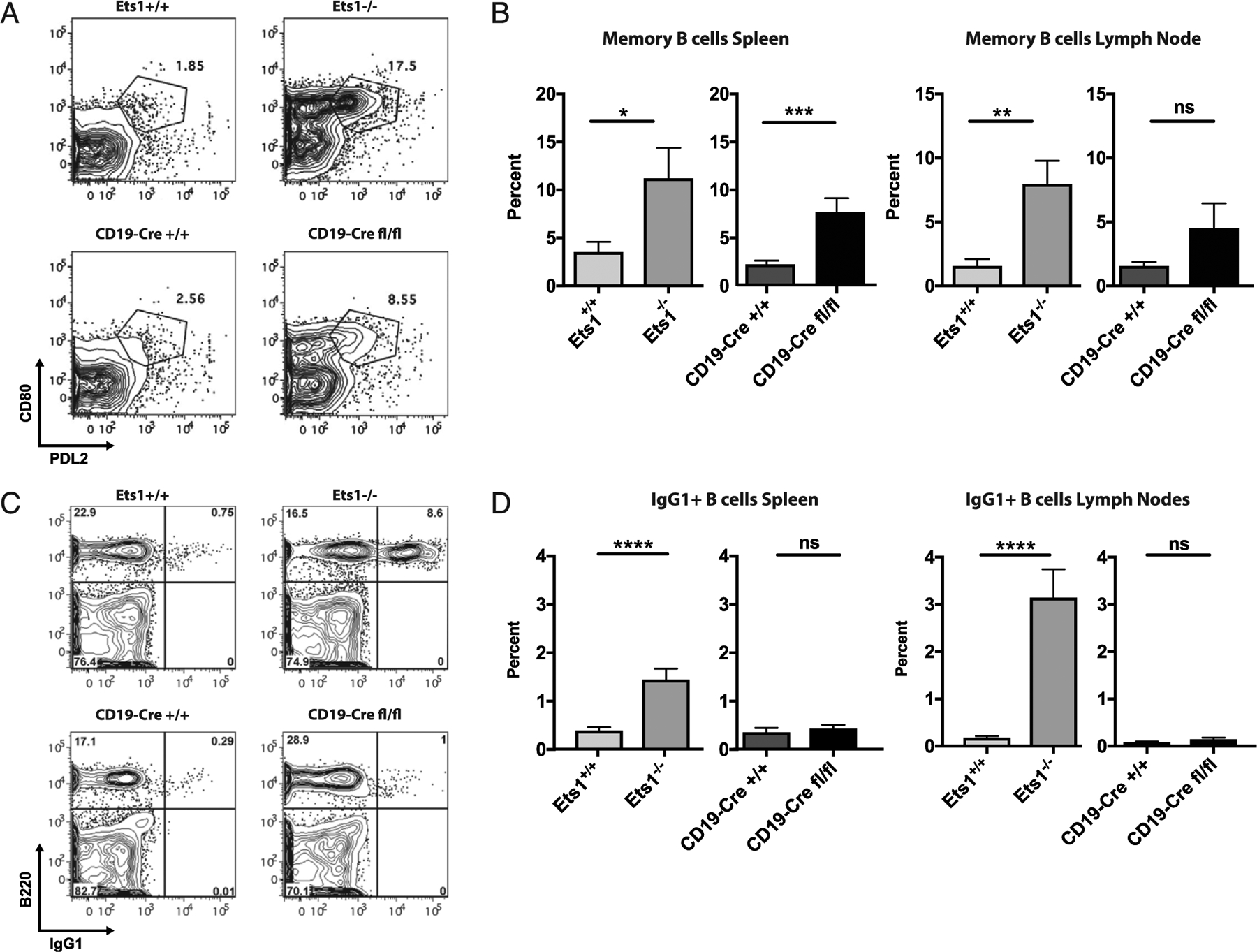
Mice with a B cell–specific deletion of Ets1 have increased memory phenotype B cells but no increase in switching to IgG1. (**A**) Flow cytometry analysis of PDL2 versus CD80 in gated live B220^+^ B cells showing that Ets1^−/−^ and CD19-Cre Ets1^fl/fl^ mice have increased percentages of B cells with a memory phenotype (PDL2^+^CD80^+^). (**B**) Quantification of the percentages of memory B cells in spleen and lymph nodes of the various strains of mice (*n* = 6 Ets1^+/+^, 6 Ets1^−/−^, 9 CD19-Cre Ets1^+/+^, and 6 CD19-Cre Ets1^fl/fl^ mice). (**C**) Flow cytometry analysis of B220 versus IgG1 in gated live splenocytes showing that Ets1^−/−^ mice, but not CD19-Cre Ets1^fl/fl^ mice, have increased percentages of IgG1^+^ B cells. (**D**) Quantification of the percentages of IgG1^+^ B cells in the spleen and lymph nodes of the various strains of mice (*n* = 21 Ets1^+/+^, 17 Ets1^−/−^, 11 CD19-Cre Ets1^+/+^, and 12 CD19-Cre Ets1^fl/fl^ mice). *p < 0.05, **p < 0.01, ***p < 0.001, ****p < 0.0001. ns, not significant.

Follicular B cells in Ets1^−/−^ mice have an activated phenotype with upregulation of CD80 and CD86. A significant fraction of the CD80+ B cells in Ets1^−/−^ mice coexpress PDL2 and CD73 (Fig. 3A and data not shown) and hence have a memory B cell phenotype, as defined previously (29). CD19-Cre Ets1^fl/fl^ mice also have an increase in B cells with a memory phenotype (CD80^+^PDL2^+^) in the spleen but not lymph nodes (Fig. 3A, 3B). In contrast, there was no increase of CD86 staining on B cells from CD19-Cre Ets1^fl/fl^ mice (Supplemental Fig. 2A, 2B).

A significant fraction of B cells in spleens and lymph nodes of conventional Ets1 knockout mice express surface IgG1 (Fig. 3C, 3D). However, very few B cells in CD19-Cre Ets1^fl/fl^ mice switch to IgG1. Class-switching typically occurs in the germinal center, and conventional Ets1 knockout mice have increased percentages of B cells with a germinal center phenotype (B220^+^PNA^+^FAS^+^) in their lymph nodes but not spleens. CD19-Cre Ets1^fl/fl^ mice do not show an increase in the numbers of GC B cells (Supplemental Fig. 2C, 2D). In summary, specific deletion of Ets1 in B cells alone results in the loss of the marginal zone B cell population and an increase in ASCs and memory B cells in the spleen but not lymph nodes. However, B cell–specific deletion of Ets1 is not sufficient to induce class-switchingtoIgG1ortheformation of germinal center B cells.

### T cells do not show an activated phenotype in CD19-Cre Ets1^fl/fl^ mice

**FIGURE 4.**
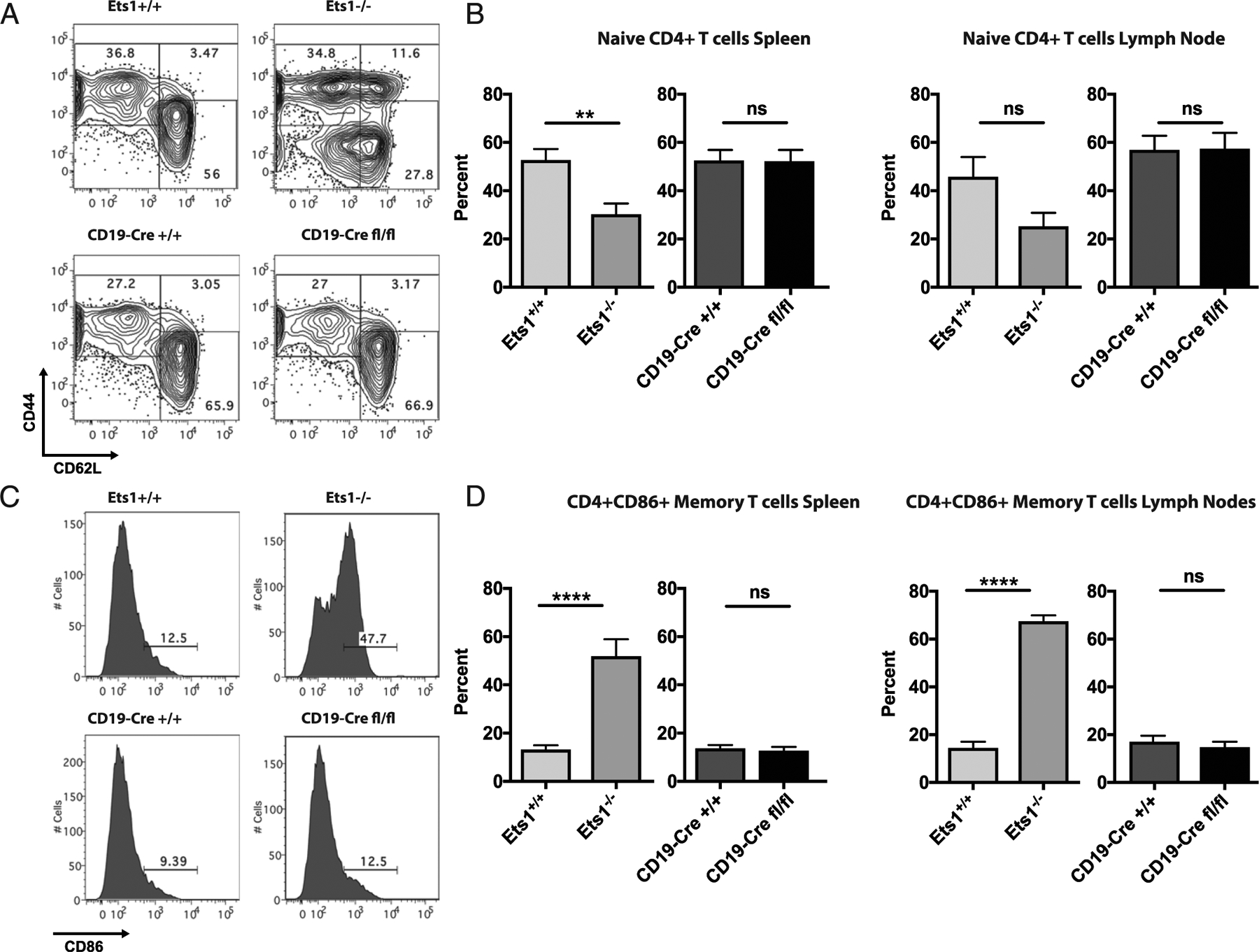
Mice with a B cell–specific deletion of Ets1 do not have increased CD4^+^ T cell activation. (**A**) Flow cytometry analysis of CD62L versus CD44 in gated live CD4^+^ T cells in spleen of the indicated mice. (**B**) Quantification of the percentages of naive phenotype CD4 T cells (CD62L^hi^CD44^lo^) in spleen and lymph nodes of the various strains of mice (*n* = 7 Ets1^+/+^, 5 Ets1^−/−^, 10 CD19-Cre Ets1^+/+^, and 8 CD19-Cre Ets1^fl/fl^ mice). (**C**) Flow cytometry analysis of CD86 staining on CD4^+^ T cells of mice. (**D**) Quantification of the percent of CD4^+^ CD86^+^ T cells in gated live spleen and lymph node cells of the indicated mice (*n* = 7 Ets1^+/+^, 5 Ets1^−/−^, 10 CD19-Cre Ets1^+/+^, and 8 CD19-Cre Ets1^fl/fl^ mice). **p < 0.01, ****p < 0.0001. ns, not significant.

Both CD4^+^ and CD8^+^ T cells from conventional Ets1^−/−^ mice have an activated phenotype (9). B cells can act as APCs to T cells, resulting in T cell activation (30–34). Therefore, it is possible that spontaneous activation of B cells in CD19-Cre Ets1^fl/fl^ mice could result in the secondary activation of the T cells. To test this possibility, markers of T cell activation were examined on cells isolated from CD19-Cre Ets1^fl/fl^ mice. In CD19-Cre Ets1^fl/fl^ mice, the majority of CD4^+^ T cells are of the naive phenotype, whereas CD4+ T cells are skewed toward an activated effector/memory phenotype in Ets1^−/−^ mice (Fig. 4A, 4B). T cells from conventional Ets1^−/−^ mice upregulate expression of CD86 on their surfaces (Fig. 4C, 4D) (9). CD86 expression is found on memory T cells after their interaction with dendritic cells (35–37), suggesting that the CD86^+^ T cells in Ets1^−/−^ mice may represent memory T cells. CD19-Cre Ets1^fl/fl^ mice do not upregulate CD86 on T cells, suggesting that they do not have an increase in memory phenotype T cells (Fig. 4C, 4D).

Impaired Treg activity has also been implicated in driving the changes in B cells lacking Ets1 (8). A normal percentage of CD4^+^ T cells in spleens and lymph nodes of Ets1^−/−^ mice expressed Foxp3 (Supplemental Fig. 3A–C), unlike what has previously been reported (8). In fact, the percentage of Foxp3^+^ cells within the CD4^+^ gate was slightly increased in Ets1^−/−^ mice. However, the overall intensity of Foxp3 staining was reduced in Tregs from the lymph nodes of Ets1^−/−^ mice (Supplemental Fig. 3D). Mice with a B cell–specific knockout of Ets1 showed similar percentages of Foxp3^+^ CD4^+^ T cells as wild-type mice and no decrease in the intensity of Foxp3 staining. Therefore, the B cell activation found in CD19-Cre Ets1^fl/fl^ mice is not the result of nor does it lead to secondary activation of the T cell population or changes in the Treg population.

### Loss of Ets1 specifically in B cells leads to formation of autoantibodies

**FIGURE 5.**
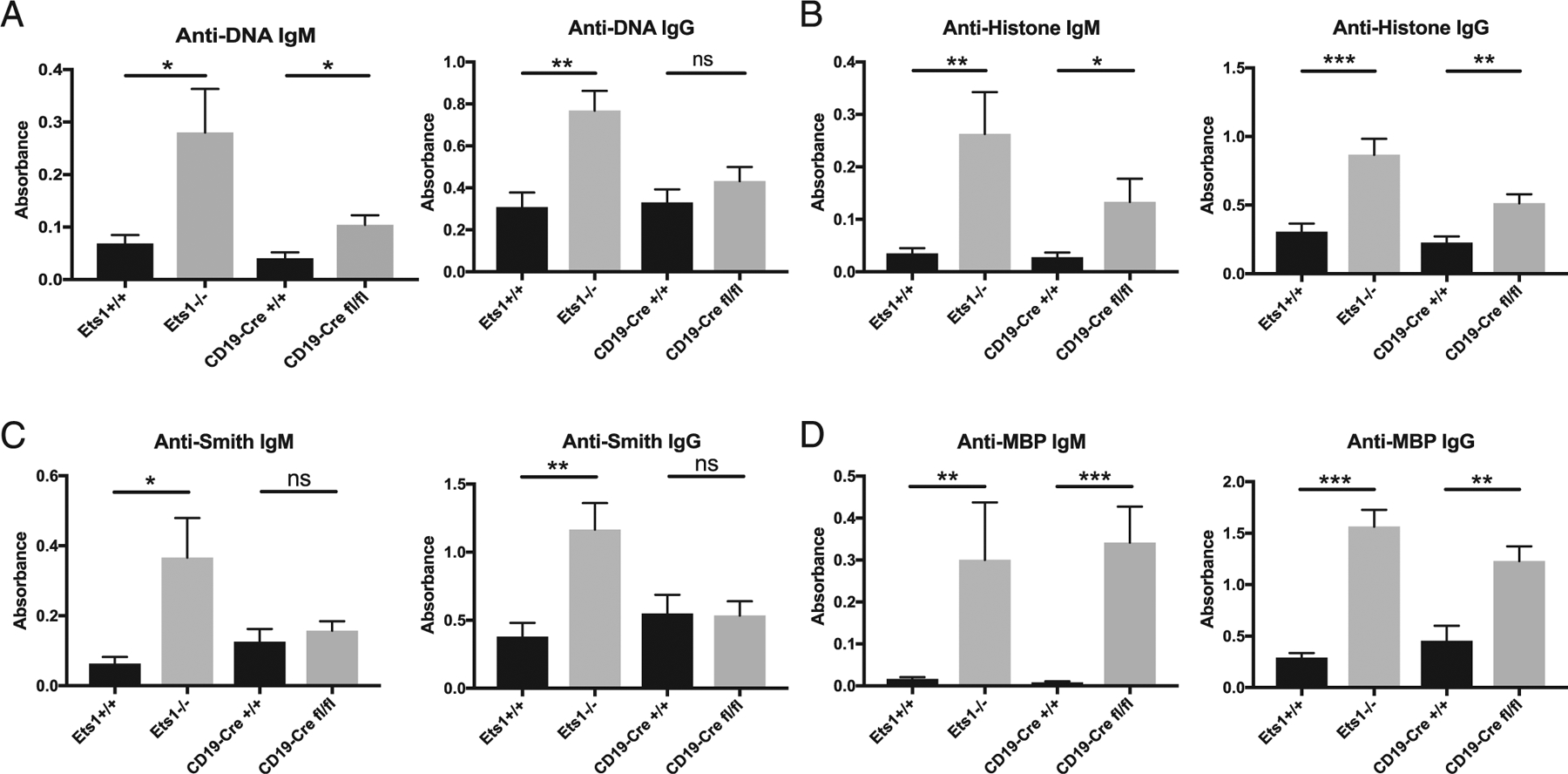
Mice with a B cell–specific deletion of Ets1 produce IgM and IgG autoantibodies. ELISA quantification of IgM and IgG autoantibodies against (**A**) dsDNA (*n* = 7 Ets1^+/+^, 7 Ets1^−/−^, 7 CD19-Cre Ets1^+/+^, and 9 CD19-Cre Ets1^fl/fl^ mice), (**B**) mixed histone proteins (H2A, H2B, H3, and H4) (*n* = 8 Ets1^+/+^, 8 Ets1^−/−^, 7 CD19-Cre Ets1^+/+^, and 8 CD19-Cre Ets1^fl/fl^ mice), (**C**) Smith Ag (*n* = 6 Ets1^+/+^, 6 Ets1^−/−^, 6 CD19-Cre Ets1^+/+^, and 6 CD19-Cre Ets1^fl/fl^ mice), or (**D**) MBP (*n* = 7 Ets1^+/+^, 8 Ets1^−/−^, 7 CD19-Cre Ets1^+/+^, and 10 CD19-Cre Ets1^fl/fl^ mice). *p < 0.05, **p < 0.01, ***p < 0.001. ns, not significant.

As described above, there is increased B cell differentiation to ASCs in CD19-Cre Ets1^fl/fl^ mice. This finding was correlated with a trend toward increased levels of serum IgM in CD19-Cre Ets1^fl/fl^ mice, although this was not statistically significant (Table I). Serum IgG was not elevated in CD19-Cre Ets1^fl/fl^ mice (Table I). IgM and IgG autoantibodies were also examined. As shown in Fig. 5A–D, Ets1^−/−^ mice showed elevated levels of both IgM and IgG autoantibodies for dsDNA, histone proteins, Smith Ag, and MBP. In contrast, CD19-Cre Ets1^fl/fl^ mice have elevated IgM and IgG autoantibodies against histones and MBP and elevated IgM autoantibodies against DNA. However, unlike Ets1^−/−^ mice, CD19-Cre Ets1^fl/fl^ mice lack autoantibodies against the RNA-associated Smith Ag.

**FIGURE 6.**
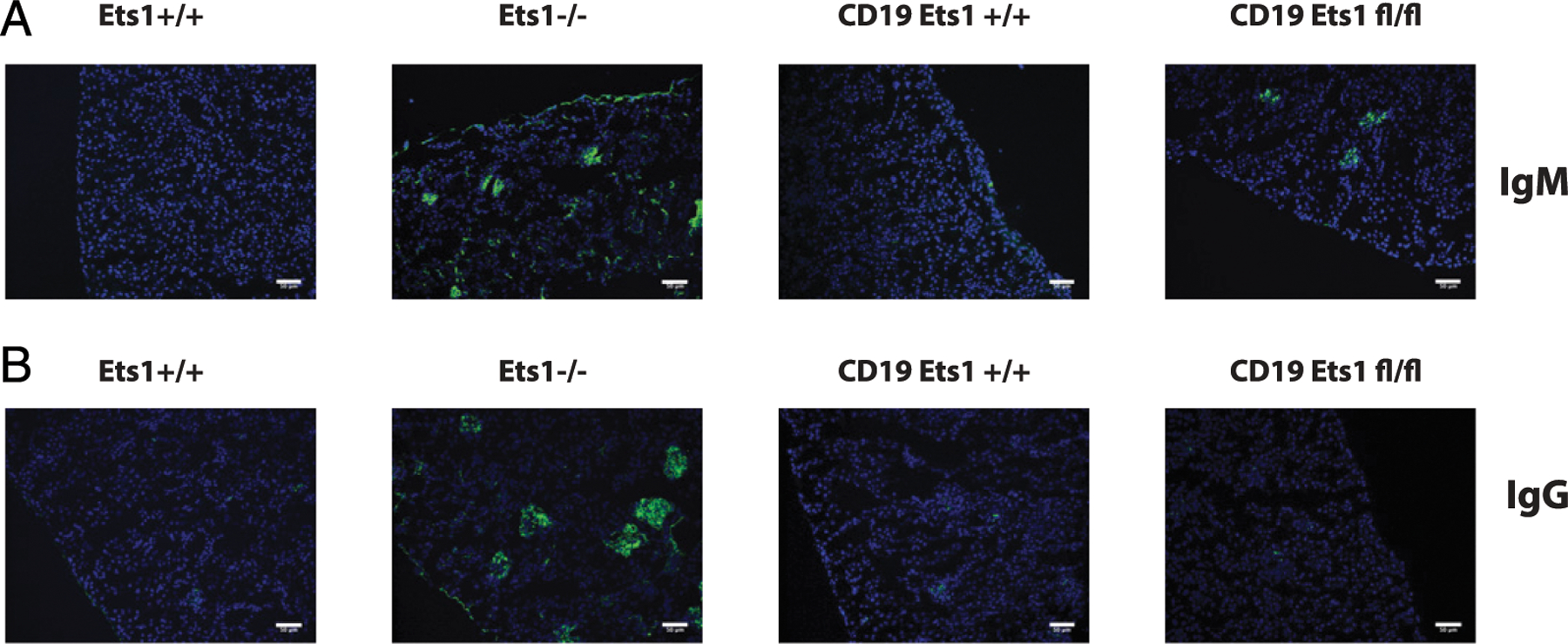
CD19-Cre Ets1^fl/fl^ mice have IgM immune complexes that deposit in the kidneys. Immunostaining of kidney sections with FITC-labeled (green) anti-mouse IgM (**A**) or anti-mouse IgG (**B**). Nuclei are counterstained with DAPI (blue) (*n* = 4 mice of each genotype for each Ab isotype). All scale bars are 50 μm.

To further confirm the production of autoantibodies, immune complex deposition in the kidneys of mice was examined. As shown in Fig. 6A and 6B, kidneys fromEts1^−/−^mice have both IgM and IgG autoantibody deposits. In contrast, CD19-Cre Ets1^fl/fl^ mice showed similar deposition of IgM immune complexes, whereas deposition of IgG immune complexes was comparatively weak.

## DISCUSSION

The *Ets1* gene is a crucial regulator of immune cell functions and a susceptibility locus for numerous autoimmune and inflammatory conditions. Ets1 is expressed at high levels in both B and T lymphocytes and at lower levels in other cell types. Mice with a conventional deletion of the *Ets1* gene, where Ets1 is absent in all cell types, develop an autoimmune syndrome similar to lupus (4, 8, 20), with increased activation of both B and T cells. A recent study has shown that deletion of Ets1 specifically in T cells results in severe autoimmune symptoms that mimic complete deletion of the gene (20). Ets1 was shown to suppress development of Tfh2 cells that secrete IL-4. The increases in Tfh2 cells drive B cell activation with development of germinal centers and increased isotype switching, ASC generation, and secretion of autoantibodies. In the same study, a B cell-specific knockout of Ets1 using CD19-Cre was also reported, although not extensively characterized (20). In fact, the only phenotype reported for the B cell deletion of Ets1 was increased serum IgM (20). In this study, deletion of Ets1 in B cells was shown to lead to numerous additional cell-intrinsic changes in the B cell compartment, including decreased percentages of marginal zone B cells, increased numbers of Ab-secreting plasma cells, increased numbers of B cells with a memory phenotype, and increased titers of autoantibodies. These changes are achieved without any apparent hyperactivation of the T cell compartment. Mice with a conventional deletion of the Ets1 gene, in which Ets1 is lacking in all tissues, show an increase in germinal center B cells and switching to IgG1. However, this phenotype is not found in mice with a B cell–specific deletion of Ets1. B cells from conventional Ets1 knockout mice also show upregulation of CD23 and CD86, which are not upregulated on B cells from CD19-Cre Ets1^fl/fl^ mice. These phenotypes are likely driven by Ets1-deficient autoreactive T cells. Some parameters controlled by Ets1, such as the development of ASCs and memory B cells and the production of autoantibodies, have a B cell–intrinsic component but show a more significant deviation from normal in conventional Ets1 knockout mice than in B cell–specific Ets1 knockout mice. This suggests that Ets1 plays roles in both B and T cells to control these differentiation steps.

Ets1^−/−^ mice produce IgM and IgG autoantibodies against a wide range of Ags including DNA, histones, Smith Ag, and MBP. CD19-Cre Ets1^fl/fl^ mice produce only a subset of these autoantibodies. For instance, both IgM and IgG autoantibodies were detected against histone proteins and MBP, whereas only IgM autoantibodies were found that recognized dsDNA. In contrast, neither IgM nor IgG autoantibodies against the RNA-associated Smith Ag were found in CD19-Cre Ets1^fl/fl^ mice. These observations hint that B cells may be activated in different ways in conventional Ets1^−/−^ mice versus CD19-Cre Ets1^fl/fl^ mice. One possibility is that autoantibodies that develop in CD19-Cre Ets1^fl/fl^ mice may be derived from T cell–independent activation of B cells, whereas T cell–dependent responses may predominate in Ets1^−/−^ mice. Costimulation of B cells via nucleic acid–specific TLRs (e.g., TLR9) may stimulate autoantibody production against DNA or histone proteins. However, if TLR signaling is involved in autoantibody production in CD19-Cre Ets1^fl/fl^ mice, it seems not to be sufficient to costimulate autoantibody production against the RNA-associated Smith Ag, despite the fact that this autoantigen signals via TLR7 (38, 39). An alternate T cell–independent pathway might involve T-independent type II activation of B cells. This may be the case for Ags present in repetitive structures, such as the polymerized nucleotides in the DNA, the histone subunits in nucleosomes of chromatin, or the network of repetitive subunits on the cytoplasmic face of the myelin membrane formed by MBP. Death of cells may lead to exposure of these repetitive elements, which could potentially extensively cross-link the BCR of Ag-specific B cells and activate them in the absence of T cell help.

It is also possible that T cells could provide help to B cells from CD19-Cre Ets1^fl/fl^ mice, leading to their activation and ASC differentiation. Although the bulk population of T cells did not show an activated phenotype and Tregs were present in normal numbers, it remains possible that a small population of Ag-specific T cells become activated and coordinate B cell responses. If so, they most likely drive extrafollicular B cell responses, given that few germinal center B cells were found in CD19-Cre Ets1^fl/fl^ mice. The lack of germinal center B cells in CD19-Cre Ets1^fl/fl^ mice is somewhat at odds with increased formation of memory B cells (CD80^+^PDL2^+^), which are typically thought to be derived from germinal centers. However, T cell–independent extrafollicular B cell responses can also promote memory B cell formation (40, 41).

Previously, Ets1^−/−^ mice were reported to harbor reduced numbers of Foxp3^+^ Tregs (8). In this study, no reduction in the numbers of Foxp3^+^ Tregs in spleen or lymph nodes of either conventional Ets1 knockout mice or B cell–specific Ets1 knockout mice was observed. However, CD4^+^ T cells from the lymph nodes of conventional Ets1 knockout mice expressed low levels of Foxp3. This is consistent with previous studies, which demonstrate that Ets1 can directly regulate the expression of the Foxp3 gene itself (8, 42). The lower than normal levels of Foxp3 in Tregs of conventional Ets1 knockout mice may contribute to the immune aberrations in those animals. However, because Foxp3 expression is normal in CD19-Cre Ets1^fl/fl^ mice, this cannot explain B cell phenotypes in these mice.

In humans, single nucleotide polymorphisms in the *ETS1* gene locus have been associated with multiple autoimmune and inflammatory diseases including lupus, rheumatoid arthritis, psoriasis, and atopic dermatitis (43). A number of studies have also shown low levels of Ets1 mRNA in PBMCs of patients with autoimmune disease (12, 13, 16, 44). Given the B cell–intrinsic roles for Ets1 described in this article, low expression of Ets1 in human B cells could result in increased differentiation and Ab secretion. Low Ets1 levels have also been described in purified T cells from autoimmune patients (45, 46) and could also promote disease pathogenesis. Therefore, genetic changes that reduce Ets1 expression in B cells or T cells or both may contribute to the development of an autoimmune response.

## Supplementary Material

1

## Figures and Tables

**TABLE I.** Serum IgM and IgG levels

	Ets1^+/+^	Ets1^−/−^	CD19-Cre Ets1^+/+^	CD19-Cre Ets1^fl/fl^
Total serum IgM (μg/ml)	347.5 ± 123	1947 ± 307***	356 ± 204	579 ± 102
Total serum IgG (mg/ml)	6.21 ± 1.60	10.19 ± 2.19	6.29 ± 2.24	4.21 ± 0.94

*** *p* < 0.001 (comparing Ets1^−/−^ to Ets1^+/+^ for serum IgM; all other differences are not statistically significant).

## Abbreviations used in this article:

ASC: Ab-secreting cell
MBP: myelin basic protein
Tfh2: T follicular helper type 2
Treg: regulatory T cell
