# Supplementary material for: Ets1 Controls the Development of B Cell Autoimmune Responses in a Cell-Intrinsic Manner
Source: Immunohorizons. Author manuscript; Available in PMC 2020 Feb 10. (PMC7008956; doi:10.4049/immunohorizons.1900033)
Supplement: 1 [file NIHMS1552542-supplement-1.pdf]

Supplemental Figure 1

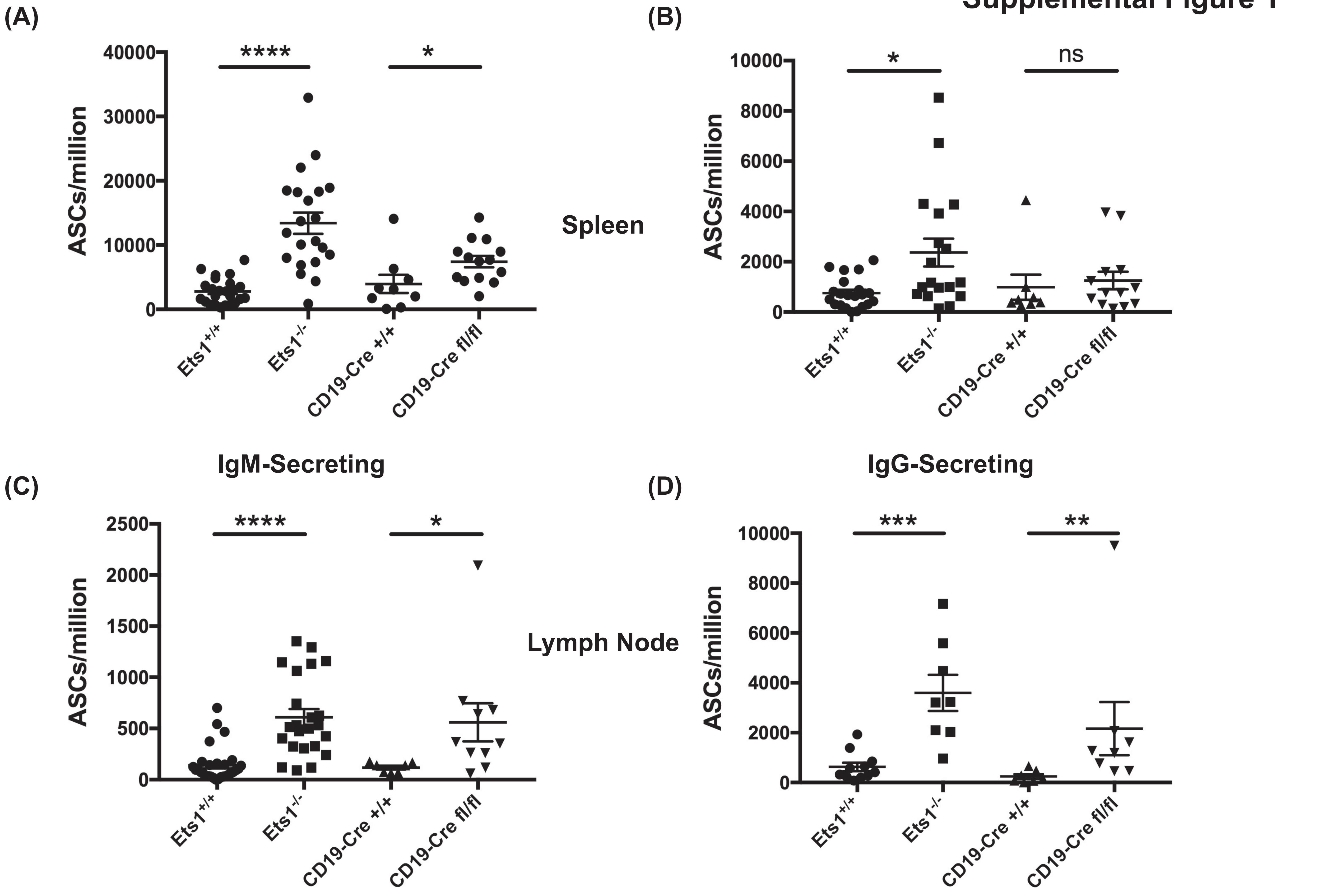

Supplemental Figure 1. CD19-Cre Ets1fl/fl mice have increased numbers of ASCs by ELISPOT. Quantification of IgM and IgG antibody-secreting cells in spleen and lymph nodes using ELISPOT. (Top) Spleen cells . (Bottom) Lymph Node cells (n 7-20= mice). (n=20 Ets1+/+, 20 Ets1-/-, 9 CD19-Cre Ets1+/+ and 14 CD19-Cre Ets1 fl/fl mice) -*p<0.05; **p<0.01; ***p<0.001; ****p<0.0001; ns – not significant.

Supplemental Figure 2

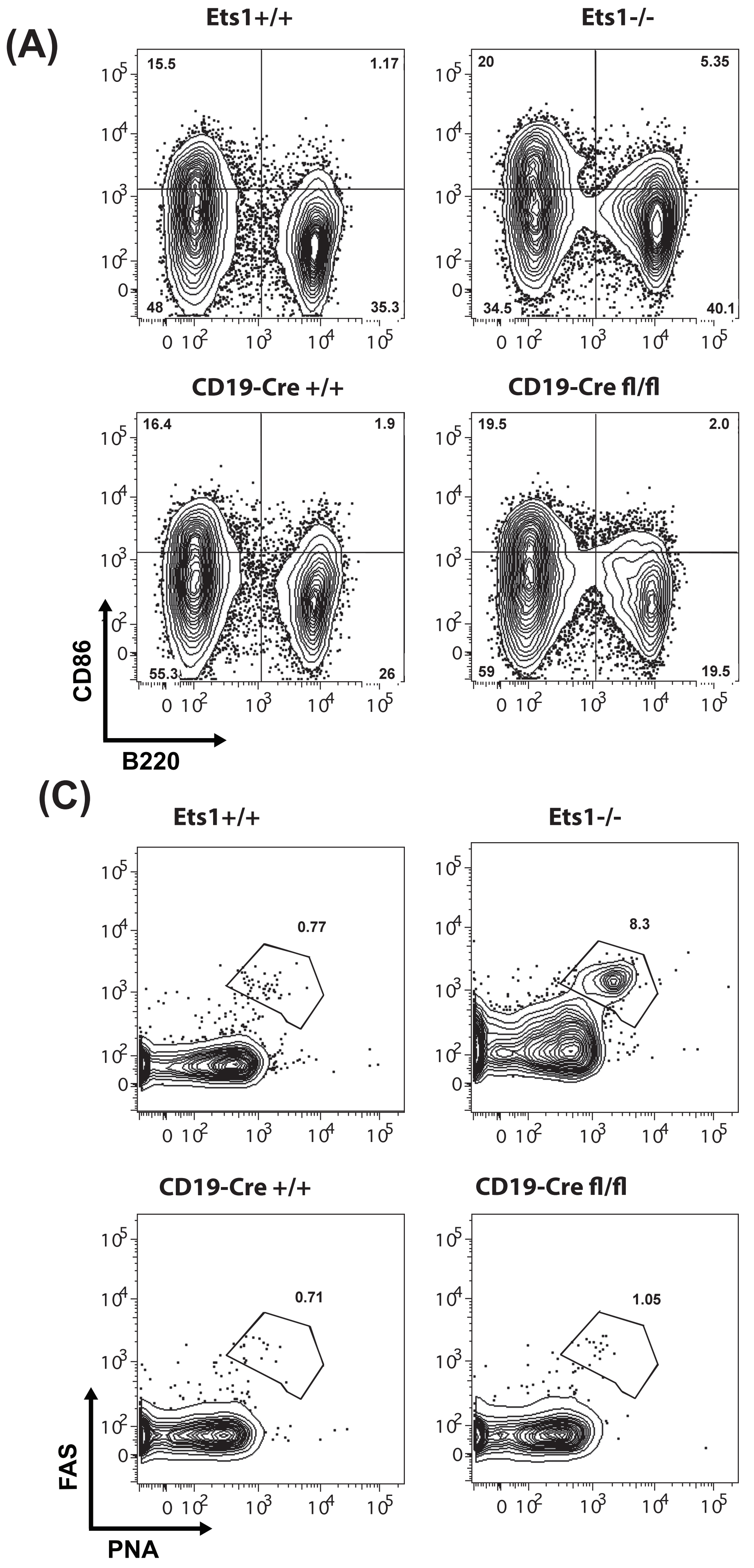

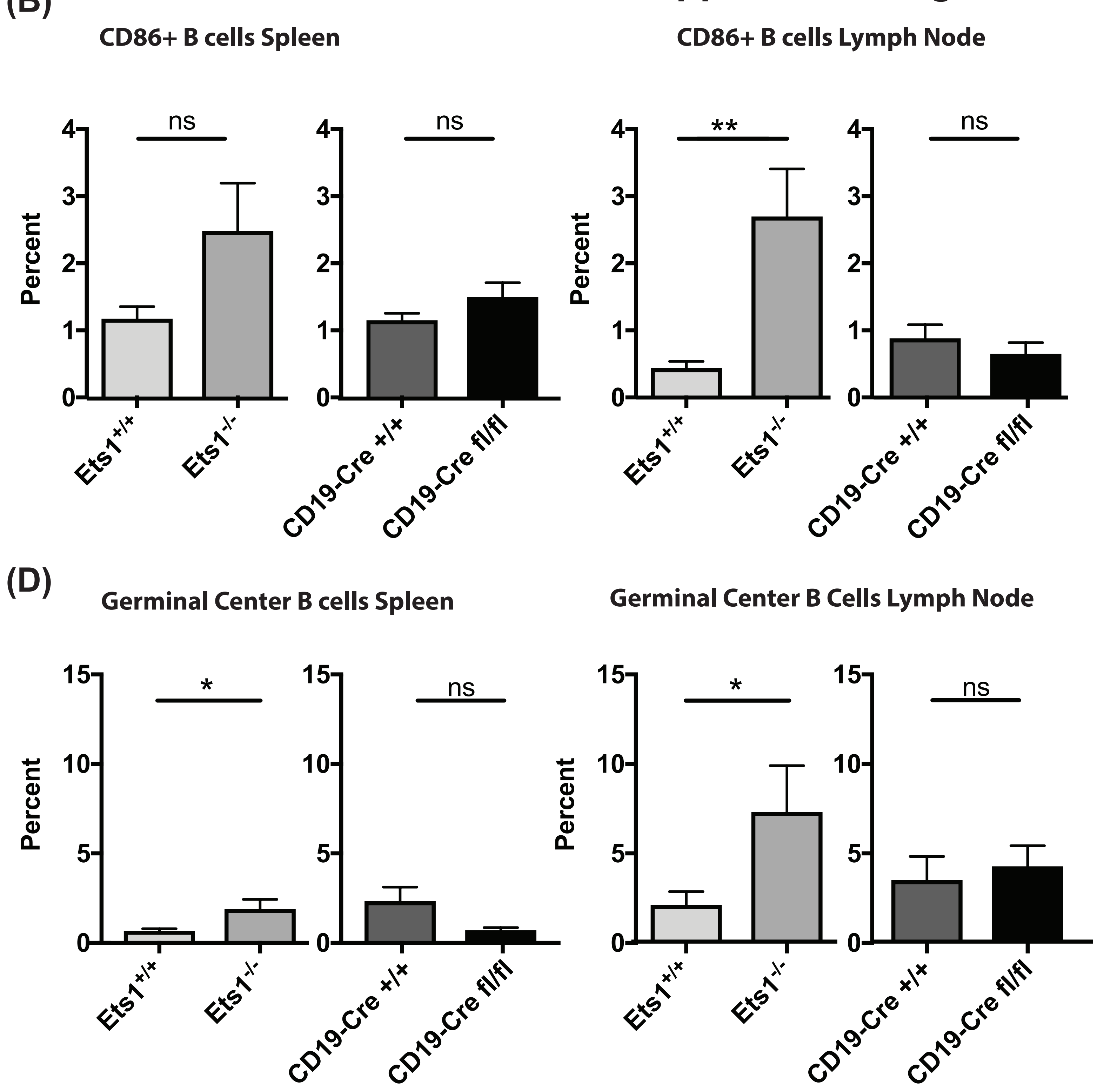

Supplemental Figure 2. Ets1-/- mice, but not CD19-Cre Ets1fl/fl mice, have increases in CD86+ B cells and germinal center B cells. (A) Flow cytometry analysis of B220 versus CD86 in gated live spleen cells showing that Ets1-/- mice, but not CD19-Cre Ets1fl/fl mice, have increased percentages of CD86+ B cells. (B) Quantification of the percentages of CD86+ B cells in spleen and lymph nodes of the various strains of mice (n=8 Ets1+/+, 6 Ets1-/-, 11 CD19-Cre Ets1+/+ and 8 CD19-Cre Ets1 fl/fl mice). (C) Flow cytometry analysis of PNA versus Fas staining in gated live, B220+ spleen cells showing that Ets1-/- mice, but not CD19-Cre Ets1fl/fl mice, have increased percentages of germinal center B cells. (D) Quantification of the percentages of germinal center B cells in the spleen and lymph nodes of the various strains of mice (n=10 Ets1+/+, 8 Ets1-/-, 11 CD19-Cre Ets1+/+ and 8 CD19-Cre Ets1 fl/fl mice). *p<0.05; **p<0.01; ns – not significant.

Supplemental Figure 3

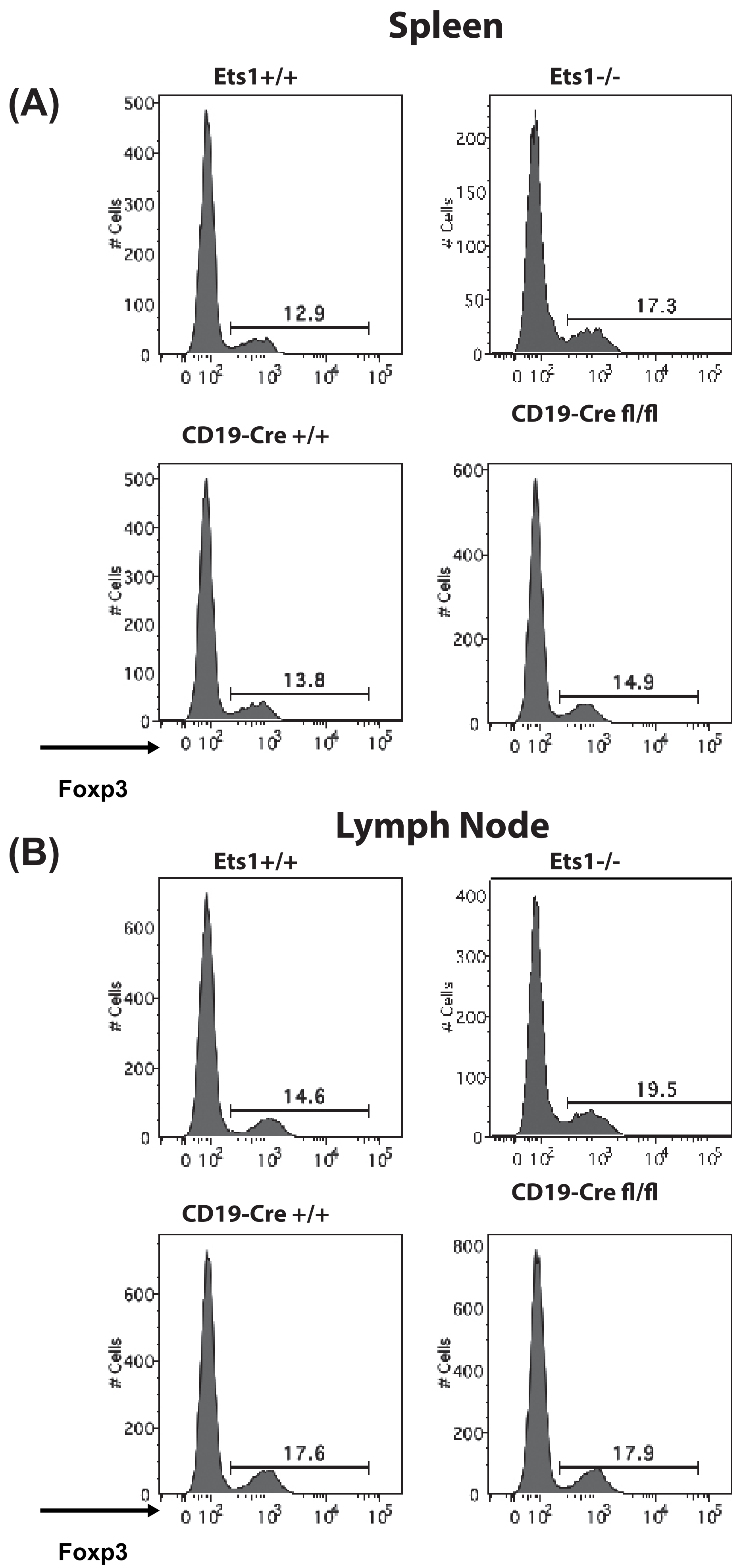

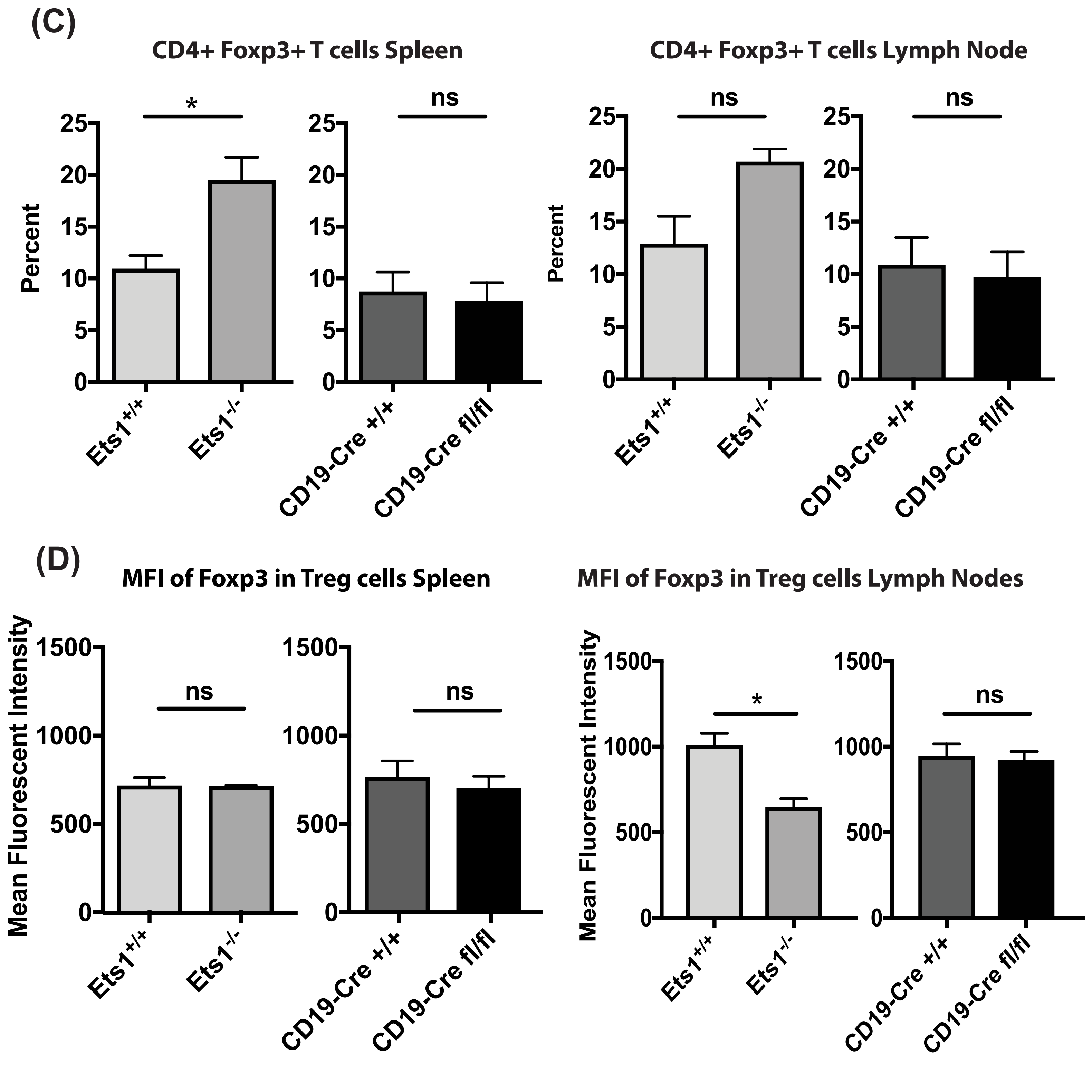

Supplemental Figure 3. Ets1-/- mice have increased percentages of CD4+Foxp3+ regulatory T cells, although the intensity of Foxp3 staining is reduced in Tregs from lymph nodes of Ets1-/- mice. (A-B) Flow cytometry analysis of Foxp3 staining in gated live CD4+ spleen and lymph node T cells. (C) Quantification of the percentages of Foxp3+ CD4 T cells in spleen and lymph nodes of the various strains of mice (n=3 Ets1-/- mice and 5 mice of the other genotypes). (D) Quantification of the mean fluorescent intensity (MFI) of Foxp3 staining in live CD4+Foxp3+ T cells in the spleen and lymph nodes of the various strains of mice. *p<0.05; ns – not significant.
